# Healthcare workers’ self-regulatory eating behaviours are associated with being stress-free during the Covid-19 lockdown in Singapore

**DOI:** 10.1038/s41598-022-19001-1

**Published:** 2022-09-28

**Authors:** Zhongwei Huang, Pei Ting Tan, Zaylea Kua, Li Jiuen Ong, Fadzil Bin Mohamed Hamzah, Benedict Tan

**Affiliations:** 1 Institute of Molecular and Cell Biology, Agency of Science, Technology and Research, Singapore, Singapore; 2 Department of Obstetrics & Gynaecology, National University Health Systems, NUHS Tower Block Level 12, 1E Kent Ridge Road, Singapore, 119228 Singapore; 3 Department of Physiology, Yong Loo Lin School of Medicine, National University of Singapore, Singapore, Singapore; 4 Clinical Trials & Research Unit, Changi General Hospital, Singapore, Singapore; 5 Department of Psychological Medicine, Changi General Hospital, Singapore, Singapore; 6 Department of Dietetic and Food Services, Changi General Hospital, Singapore, Singapore; 7 Department of Sport & Exercise Medicine, Changi General Hospital, Singapore, Singapore

**Keywords:** Psychology, Health care, Health occupations, Risk factors

## Abstract

Our study sought to examine the impact of the pandemic and the Circuit-breaker (CB) measures on dietary behaviours of healthcare workers (HCW). In addition, the association between self-regulatory eating behaviours and psychological stress was explored. Our study employed a cross-sectional anonymous survey that examined the demographics, dietary habits, self-regulatory eating behaviours (SR) and their association to stress levels of healthcare workers (HCWs) before and during the Circuit Breaker (CB) in Singapore**.** The survey was conducted over four weeks from May 17 to June 18, 2020. Snowball sampling was performed in the final week. A total of 707 participants took part in the survey. Due to the CB measures and modifications of work scope and work areas, there were significant changes in their dietary behaviors before CB versus during the CB period (n = 707), with many reducing the intake of vegetables (*p* = 0.018) while increasing their intake of unhealthy food choices such as canned drinks (*p* = 0.002), convenience food (*p* ≤ 0.001) and alcoholic drinks (*p* = 0.034). Before the CB period, 91.8% (602/656) of participants who intended to have a healthy diet were classified in medium-to-high SR groups whereas during the CB period, 87.7% (575/656) were in medium-to-high SR groups and the difference was statistically significant (*p* = 0.011). Nurses, administrative staff, HCWs of Chinese and Indian ethnicities, staff who did not complete university education and those who did not have domestic helpers were more likely to have reduced SR. Importantly, amongst participants who intended to have a healthy diet, 70.9% displayed no change or improved eating habits and showed medium-to-high levels of SR during CB. Participants with no changes in SR were 2.11 times more likely to be stress-free as compared to those who had deteriorated SR (OR 2.11 95% CI 1.27–3.48, *p* = 0.004). Due to CB measures and work modifications, dietary behaviours of HCWs deteriorated with increased intake of unhealthy food choices. HCWs who maintain their self-regulatory eating behaviour are more likely to be stress-free. Short screening questionnaires based on SR changes should be developed and explored as surveillance tools for assessment of HCWs’ general well-being such that personalized interventions to vulnerable groups of workers could be implemented effectively on the ground.

## Introduction

The COVID-19 pandemic has severely disrupted people’s lives. The exponential spread of the coronavirus globally has forced many countries to enforce border closures, social distancing measures, wearing of masks and eventual lockdown to slow the spread of the virus. The lockdown imposed on April 7, 2020, in Singapore, called “the Circuit Breaker (CB)”^1^, comprised measures aimed at reducing movements and interactions in public and private places. Workplaces were encouraged to implement telecommuting arrangements for employees. Non-essential sectors like sports and recreational facilities were ordered to close and eateries were opened only for takeaway or delivery. These containment measures have consequently led to changes in lifestyle choices among the general population including increased consumption of carbohydrate sources and snacking behaviours, restricted water intake, as well as concerns about weight gain and obesity^2,3^. In a multi-national cohort study, self-reported dietary quality was found to be significantly related to differences in risk of COVID-19, even after accounting for potential confounders including social determinants of health and virus transmission measures. Participants with dietary patterns consisting of healthy plant food were found to be at significantly lower risk and severity of COVID-19 than those with low quality diet^4^.

For many healthcare workers (HCWs) who were continually exposed to frontline emergency duties, emerging evidence suggests that their dietary behaviours and nutritional status may have been compromised. In China, HCWs experienced an imbalanced diet with excessive consumption of salt and oil through box meals provided, leading to changes in body weight even within a short span of 2 months^5^. In Japan, HCWs who ate less balanced meals were more likely to be working longer hours and less likely to eat with others. In particular, the researchers found a significant association between depressive symptoms and frequency of balanced meal consumption. Wherein, HCWs who consumed balanced meals for less than 3 days a week were significantly more likely to report depressive symptoms compared to those on balanced meals daily, even after controlling for lifestyle and COVID-19 related work variables^6^. In Vietnam, healthcare workers who showed greater literacy for healthy eating and engaged in healthy eating behaviours were more likely to report stable or better psychological well-being during the pandemic^7^. This calls to the attention of emphasizing optimal diet among HCWs given their susceptibility to stress and burnout during the pandemic.

Although these regulations were pertinent measures to tide over the global pandemic, many studies had examined the impact of Covid-19 lockdown on the physical activity and dietary choices in general populations^8–15^. However, limited studies focused on healthcare workers during these critical moments. The physical and psycho-social consequences of limited physical activities^15^, minimal social interactions amongst colleagues in the same department, abrupt changes in working environments, changes in sleep habits and reduced dietary choices could result in adverse effects on HCWs^16,17^, who continued to provide daily essential services during these difficult times. Psychological problems such as fear, depression and stress, along with physical restrictions, may cause changes in individuals’ eating habits or trigger emotional eating. Given the various roles HCWs had to function daily in terms of shift work and odd working hours to ensure round-the-clock coverage of healthcare facilities, the impact on their diet choices, time for meal preparation and exercise/physical activities might be compromised further. There is currently no sufficient emphasis placed on the role of nutrition against stress and anxiety among HCWs^18,19^. HCWs run the risk of making convenient and unhealthy food choices, with a high tendency to overeat once-off from work to relieve stress by means of comfort food such as fast food and snacks, energy-dense and nutrients-poor food. As a result, HCWs may be unable to restore or maintain adequate nutritional status, which is crucial to cope with continuous stress and maintain immunity during the pandemic.

Our study sought to examine the impact of the pandemic and the Circuit-breaker (CB) measures on the dietary behaviours of healthcare workers (HCWs) in Singapore. In addition, the association between self-regulatory eating behaviours and psychological stress was explored. This will allow authorities to craft health messages, devise specific dietary advice and interventions specific to healthcare workers to keep them resilient and healthy during public health crises, as they battle at the frontlines.

## Method

Our study employed a cross-sectional survey that examined physical activity, diet, and mental well-being of healthcare workers before and during the CB in Singapore. The anonymous survey was constructed using an online form builder tool known as ‘FormSG’ developed by the Government Technology Agency (GovTech) which enables the creation of encrypted governmental digital forms^20^. The multi-domain structured survey was written in English, and comprised data collected on the sociodemographic characteristics, dietary habits, physical activity levels, and psychological status of the participants.

Participants were recruited through convenience sampling via Singapore Health Services (SingHealth) Private Limited (https://www.singhealth.com.sg/), comprising of four public hospitals, three community hospitals, five national specialty centres and eight polyclinics with a total of 29,894 employees in 2020. The inclusion criteria of our study included all healthcare workers in these institutions i.e., doctors, nurses, and allied health professionals, administrative and clerical staff and they were invited to participate the online survey. The survey was opened over four weeks from May 17 to June 18, 2020, and snowball sampling was performed in the final week. Further details of recruitment methods can be found in the authors’ earlier publication^21^. Ethics approval was obtained from the SingHealth Institutional Review Board and the need for informed consent was waived by the ethics committee of SingHealth Institutional Review Board (CIRB Ref 2020/2414) as this is an online survey that is conducted anonymously. The study was carried out following all relevant guidelines and regulations.

### Sociodemographic data collection

Data were collected on age, gender, nationality, race, education level, marital status, number of children, place of residence, household members in addition to the participant’s job title, change in work location and hours of work before and during the CB in Singapore.

### Assessment of dietary practices, preferences and self-regulation of eating behaviour

We adapted questions from the validated Self-Regulation of Eating Behaviour Questionnaire (SREBQ) by Kliemann and colleagues with the corrected item-total correlation of SREBQ ranging from 0.36 to 0.65, and the Cronbach’s alpha was 0.75^22^. Our survey aimed to examine the changes before and during the circuit breaker on their intentions to have a healthy diet. We assessed self-regulation of dietary behaviours (SR) in the following items to assess how the participants motivate themselves to maintain their self-perceived healthy diets (Table 1). For those who intended to have a healthy diet (i.e., answered 'Yes' to question 3 of SREBQ), the level of SR was determined based on the scoring system detailed^22^. As SREBQ was utilized to assess one’s capability to regulate eating, its utility would be limited in individuals who did not have the intention to have a healthy diet. The capacity to self-regulate eating behaviours bridges the intention-behaviour gap to determine the SR level (whether low/medium/high) for those with an intention to eat a healthy diet (Supplementary Table S1A,B).

**Table 1:** Demographic characteristics of respondents who intended to have healthy diet (n = 656).

	N (%)
Age, mean (SD)	37.6 (10.3)
**Gender**	
Male	105 (16.0)
Female	551 (84.0)
**Nationality**	
Singaporean/SPR	538 (82.0)
Non-Singaporean/non-PR	118 (18.0)
**Race**	
Chinese	379 (57.8)
Malay	108 (16.5)
Indian	65 (9.9)
Others	104 (15.9)
**Marital status**	
Married	381 (58.1)
Never married	275 (41.9)
**Highest education**	
Non-university	130 (20.7)
University	499 (79.3)
**Do you have a domestic helper at home currently?**	
No	525 (80.0)
Yes	131 (20.0
**Staying condition**	
Living own house	321 (48.9)
Living with parents/siblings/relatives	177 (27.0)
Rented house	158 (24.1)
**Occupation**	
Doctor	59 (9.0)
Nurse	218 (33.2)
Allied health	238 (36.3)
Admin	141 (21.5)
**Number of days per week spent on exercise during CB compared to before CB**	
Reduced	247 (37.7)
Remain unchanged	304 (46.3)
Increased	105 (16.0)
**Stress**	
Yes	119 (18.1)
No	537 (81.9)
**Anxiety**	
Yes	310 (47.3)
No	346 (52.7)
**Depression**	
Yes	256 (39.0)
No	400 (61.0)

### Assessment of stress levels

Stress levels of participants during the COVID-19 CB were measured using the stress subscale of the Depression, Anxiety and Stress Scale-21 items (DASS-21). Scores of participants’ stress symptoms can be obtained by doubling the summation of items on the stress subscale. Cut-off scores of > 14 indicate the presence of stress. DASS has been validated amongst the general population, medical workers and patients^21^. The stress scale also possesses high internal consistency of α = 0.90^21^.

### Statistical analysis

The participants’ sociodemographic characteristics were expressed in mean with standard deviation (SD) for age and frequency or percentage for categorical variables. Self-regulatory eating behaviour (SR) before CB and during CB was performed using the Marginal Homogeneity test.

Change in SR was derived from SR before CB and SR during CB to account for participants’ baseline SR. The distribution difference between the change in SR and stress was assessed using χ^2^ tests. The association between change in SR and no stress was assessed using multivariate binary logistic regression to adjust for possible confounders such as age, gender, nationality, marital status, having domestic helper, and number of days spent on moderate-vigorous physical activity. All statistical analyses were performed using IBM SPSS version 23.0 (IBM Corp., Armonk, NY, USA) and statistical significance was set at *p* < 0.05.

## Results

**Figure 1:**
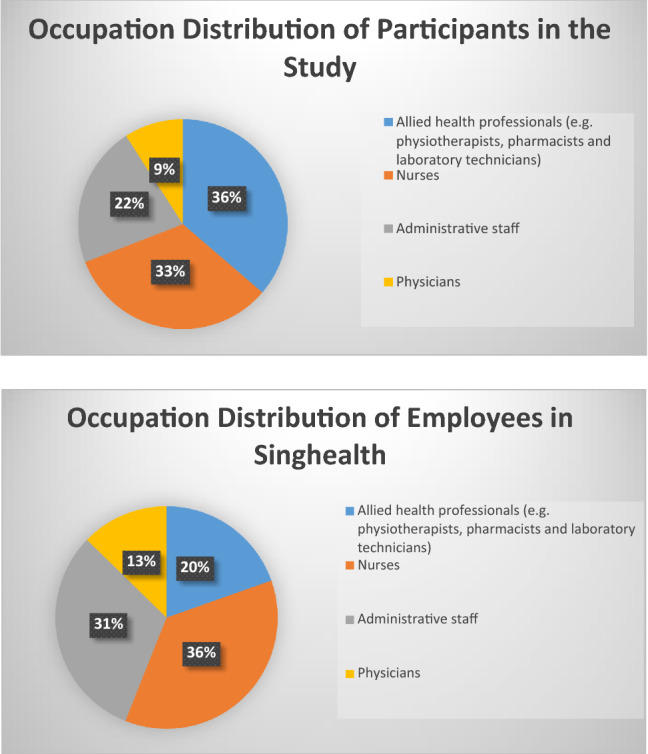
Occupation distribution of participants in the study is similar to the occupation distribution of employees in Singhealth.

Based on the dissemination methods, a total of 707 responses were collected from participants aged 17 to 78 years old with the majority being women (83.2%), Singaporean (82.7%), of Chinese ethnicity (59.0%), married (55.3%) and had attained university-level education (78%). The proportion of participants’ occupations was representative of the occupational distribution of the healthcare workforce (Fig. 1). Most of the participants continued working in their regular place of work (76.8%), while 12.5% were deployed to new work roles and 10.6% worked remotely from their homes during the CB. About half the participants reported spending the same amount of time at the workplace before and during the CB. 27.4% reported reductions in hours spent physically at the workplace while 15.8% reported an increase in working hours during CB.

### Dietary behaviours of 656 healthcare workers who intended to have a healthy diet before versus during the CB period and demographic risk factors

Of the 707 participants, 92.8% (656/707) intended to have a healthy diet (Table 2). Even amongst those who intended to have a healthy diet (n = 656), decreased vegetable intake (*p* = 0.027), increased intake of soft/canned drinks (*p* = 0.003), convenience food (*p* < 0.001) and canned/salted food (*p* < 0.001) (Tables 3, 4, 5) were observed during the CB period. It was also observed that an increased proportion of HCWs were cooking more often during CB and buying more often before the CB (*p* < 0.001). Interestingly, we observed that Chinese participants were twice more likely (OR 2.15 95% CI 1.04–4.46 *p* = 0.039) and Indian participants were 3.5 times (OR 3.54 95% CI 1.43–8.74 *p* = 0.006) more likely to have reduced SR in their dietary behaviours compared to Malay participants. Additionally, administrative staff was 2.5 times more likely to have reduced SR in their dietary behaviours as compared to nurses (OR 2.48 95% CI 1.02–6.06, *p* = 0.046) possibly reflecting the differences in work arrangements, especially where administrative staff was primarily working from home while nurses were at the frontline in the hospitals. Participants who had completed university education were 2.8 times more likely to have improved SR in their dietary behaviours as compared to those without university education (OR 2.79 95% CI 1.08–7.19 *p* = 0.034). It was found that participants who had a domestic helper were 2.3 times more likely to have improved SR in their dietary behaviours as compared to those without domestic helper (OR 2.30 95% CI 1.01–5.27, *p* = 0.048).

**Table 2:** Dietary behaviours of 656 healthcare workers who intended to have a healthy diet.

	N (%)
**Eating habits during CB duration**	
Got better/improved	117 (17.8)
No change	379 (57.8)
Got worse	150 (22.9)
Others	10 (1.5)
**For home cooked meal: who prepare the meals?**	
I prepare my own meals	415 (63.3)
My helper prepares the meals	100 (15.2)
My parents prepare the meals	226 (34.5)
My spouse prepares the meals	101 (15.4)
Prepared by others	15 (2.3)
Not applicable	22 (3.4)
**If buy outside meals: how/where do they buy their food from?**	
Walk to the franchise for takeaway	378 (57.6)
Take public transport for takeaway	101 (15.4)
Order online for food delivery services	408 (62.2)
My helper walks to the franchise for takeaway	13 (2.0)
Drive to the franchise for takeaway	152 (23.2)
Others	11 (1.1)
Not applicable	23 (3.5)
**It is important to have a healthy and balanced diet**	
Strongly disagree	1 (0.2)
Slightly disagree	0 (0.0)
Neutral	21 (3.2)
Slightly agree	59 (9.0)
Strongly agree	575 (87.7)
**It is important to choose food with less sugar**	
Strongly disagree	0 (0.0)
Slightly disagree	0 (0.0)
Neutral	30 (4.6)
Slightly agree	86 (13.1)
Strongly agree	540 (82.3)
**It is important to choose food with less fat**	
Strongly disagree	1 (0.2)
Slightly disagree	497 (75.8)
Neutral	43 (6.6)
Slightly agree	111 (16.9)
Strongly agree	497 (75.8)
**It is important to choose food with less salt**	
Strongly disagree	0 (0.0)
Slightly disagree	5 (0.8)
Neutral	34 (5.2)
Slightly agree	110 (16.8)
Strongly agree	507 (77.3)
**It is important to have an adequate intake of fibre**	
Strongly disagree	0 (0.0)
Slightly disagree	0 (0.0)
Neutral	26 (4.0)
Slightly agree	87 (13.3)
Strongly agree	543 (82.8)
**Do you intend to have a healthy diet?**	
No	51 (17.2)
Yes	656 (92.8)
**Self-regulation before CB**	
Low	54 (8.2)
Medium	429 (65.4)
High	173 (26.4)
**Self-regulation after CB**	
Low	81 (12.3)
Medium	428 (65.2)
High	147 (2.4)

**Table 3:** Food frequency *before* circuit breaker (CB).

	Food frequency *before* CB N (%)				
	Never or less than once per month	At least 1 serving per week	> 2 to 6 servings per week	Once a day	> 2 servings per day
Fruit intake	24 (3.7)	180 (27.4)	267 (40.7)	125 (19.1)	60 (9.1)
Vegetable intake	20 (3.0)	58 (8.8)	255 (38.9)	119 (18.1)	204 (31.1)
Wholegrain	113 (17.2)	212 (32.3)	178 (27.1)	98 (14.9)	55 (8.4)
Soft/canned drinks	250 (38.3)	272 (41.7)	93 (14.2)	26 (4.0)	12 (1.8)
Tea/coffee	146 (22.3)	134 (20.4)	131 (20.0)	177 (27.0)	68 (10.4)
Chocolate	217 (33.1)	329 (50.2)	90 (13.7)	15 (2.3)	5 (0.8)
Cakes/kueh	198 (30.2)	382 (58.2)	70 (10.7)	5 (0.8)	1 (0.2)
Deep fried food	67 (10.2)	353 (53.8)	206 (31.4)	23 (3.5)	7 (1.1)
Convenience food	208 (31.7)	348 (53.0)	84 (12.8)	8 (1.2)	8 (1.2)
Canned/salted food	260 (39.6)	341 (52.0)	51 (7.8)	2 (0.3)	2 (0.3)
Alcoholic drinks	485 (73.9)	135 (20.6)	33 (5.0)	2 (0.3)	1 (0.2)
Home-cooked	41 (6.3)	105 (16.0)	249 (38.0)	142 (21.6)	119 (18.1)
Buy food from outside	34 (5.2)	167 (25.5)	273 (41.6)	102 (15.5)	80 (12.2)

**Table 4:** Food frequency *during* circuit breaker (CB).

	Food frequency *during* CB N (%)				
	Never or less than once per month	At least 1 serving per week	> 2 to 6 servings per week	Once a day	> 2 servings per day
Fruit intake	29 (4.4)	179 (27.3)	272 (41.5)	108 (16.5)	68 (10.4)
Vegetable intake	25 (3.8)	67 (10.2)	253 (38.6)	108 (16.5)	203 (30.9)
Wholegrain	120 (18.3)	203 (30.9)	181 (27.6)	96 (14.6)	56 (8.5)
Soft/canned drinks	223 (34.0)	262 (39.9)	129 (19.7)	27 (4.1)	15 (2.3)
Tea/coffee	144 (22.0)	130 (19.8)	143 (21.8)	163 (24.8)	76 (11.6)
Chocolate	220 (33.5)	312 (47.6)	100 (15.2)	18 (2.7)	6 (0.9)
Cakes/kueh	204 (31.1)	349 (53.2)	96 (14.6)	6 (0.9)	1 (0.2)
Deep fried food	69 (10.5)	322 (49.1)	231 (35.2)	22 (3.4)	12 (1.8)
Convenience food	165 (25.2)	348 (53.0)	122 (18.6)	6 (0.9)	15 (2.3)
Canned/salted food	211 (32.2)	351 (53.5)	88 (13.4)	4 (0.6)	2 (0.3)
Alcoholic drinks	500 (76.2)	115 (17.5)	35 (5.3)	4 (0.6)	2 (0.3)
Home-cooked	37 (5.6)	73 (11.1)	244 (37.2)	124 (18.9)	178 (27.1)
Buy food from outside	50 (7.6)	196 (9.9)	278 (2.4)	71 (10.8)	61 (9.3)

**Table 5:** Food intake *before and during* circuit breaker (CB).

Before CB	During CB N (%)			P value
	Never or less than once per month	At least 1 to 6 serving per week	At least once a day or more	
**Fruit intake**	**N = 29**	**N = 451**	**N = 176**	**0.149**
Never or less than once per month (n = 24)	12 (1.8)	10 (1.5)	2 (0.3)	
At least 1 to 6 serving per week (n = 447)	17 (2.6)	406 (61.9)	24 (3.7)	
At least once a day or more (n = 185)	0 (0.0)	35 (5.3)	150 (22.9)	
**Vegetable intake**	**N = 25**	**N = 320**	**N = 311**	**0.027**
Never or less than once per month (n = 20)	18 (2.7)	2 (0.3)	0 (0.0)	
At least 1 to 6 serving per week (n = 313)	6 (0.9)	289 (44.1)	18 (2.7)	
At least once a day or more (n = 323)	1 (0.3)	29 (4.4)	293 (44.7)	
**Wholegrain**	**N = 120**	**N = 384**	**N = 152**	**0.359**
Never or less than once per month (n = 113)	97 (14.8)	14 (2.1)	2 (0.3)	
At least 1 to 6 serving per week (n = 390)	20 (3.0)	359 (54.7)	11 (1.7)	
At least once a day or more (n = 153)	3 (0.5)	11 (1.7)	139 (21.2)	
**Soft/canned drinks**	**N = 222**	**N = 389**	**N = 42**	**0.003**
Never or less than once per month (n = 250)	194 (29.7)	53 (8.1)	3 (0.5)	
At least 1 to 6 serving per week (n = 365)	26 (4.0)	327 (50.1)	12 (1.8)	
At least once a day or more (n = 38)	2 (0.3)	9 (1.4)	27 (4.1)	
**Tea/coffee**	**N = 144**	**N = 273**	**N = 239**	**0.713**
Never or less than once per month (n = 146)	120 (18.3)	21 (3.2)	5 (0.8)	
At least 1 to 6 serving per week (n = 265)	23 (3.5)	222 (33.8)	20 (3.0)	
At least once a day or more (n = 245)	1 (0.2)	30 (4.6)	214 (32.6)	
**Chocolate**	**N = 220**	**N = 412**	**N = 24**	**0.917**
Never or less than once per month (n = 217)	185 (28.2)	30 (4.6)	2 (0.3)	
At least 1 to 6 serving per week (n = 419)	34 (5.2)	375 (57.2)	10 (1.5)	
At least once a day or more (n = 20)	1 (0.2)	7 (1.1)	12 (1.8)	
**Cakes/kueh**	**N = 204**	**N = 445**	**N = 7**	**0.600**
Never or less than once per month (n = 198)	159 (24.2)	39 (5.9)	0 (0.0)	
At least 1 to 6 serving per week (n = 452)	45 (6.9)	403 (61.4)	4 (0.6)	
At least once a day or more (n = 6)	0 (0.0)	3 (0.5)	3 (0.5)	
**Deep fried food**	**N = 69**	**N = 553**	**N = 34**	**0.803**
Never or less than once per month (n = 67)	45 (6.9)	21 (3.2)	1 (0.2)	
At least 1 to 6 serving per week (n = 559)	24 (3.7)	526 (80.2)	9 (1.4)	
At least once a day or more (n = 30)	0 (0.0)	6 (0.9)	24 (3.7)	
**Convenience food**	**N = 165**	**N = 470**	**N = 21**	** < 0.001**
Never or less than once per month (n = 208)	140 (21.3)	65 (9.9)	3 (0.5)	
At least 1 to 6 serving per week (n = 432)	24 (3.7)	400 (61.0)	8 (1.2)	
At least once a day or more (n = 16)	1 (0.2)	5 (0.8)	10 (1.5)	
**Canned/salted food**	**N = 211**	**N = 439**	**N = 6**	** < 0.001**
Never or less than once per month (n = 260)	193 (29.4)	66 (10.1)	1 (0.2)	
At least 1 to 6 serving per week (n = 392)	18 (2.7)	371 (56.6)	3 (0.5)	
At least once a day or more (n = 4)	0 (0.0)	2 (0.3)	2 (0.3)	
**Alcoholic drinks**	**N = 500**	**N = 150**	**N = 6**	**0.140**
Never or less than once per month (n = 485)	462 (70.4)	22 (3.4)	1 (0.2)	
At least 1 to 6 serving per week (n = 168)	38 (5.8)	128 (19.5)	2 (0.3)	
At least once a day or more (n = 3)	0 (0.0)	0 (0.0)	3 (0.5)	
**Home-cooked**	**N = 37**	**N = 317**	**N = 302**	** < 0.001**
Never or less than once per month (n = 41)	25 (3.8)	12 (1.8)	4 (0.6)	
At least 1 to 6 serving per week (n = 354)	10 (1.5)	282 (43.0)	62 (9.5)	
At least once a day or more (n = 261)	2 (0.3)	23 (3.5)	236 (36.0)	
**Buy food from outside**	**N = 50**	**N = 474**	**N = 132**	** < 0.001**
Never or less than once per month (n = 34)	20 (3.0)	10 (1.5)	4 (0.6)	
At least 1 to 6 serving per week (n = 440)	21 (3.2)	402 (61.3)	17 (2.6)	
At least once a day or more (n = 182)	9 (1.4)	62 (9.5)	111 (16.9)	

It was observed that SR was statistically different before and during the CB (*p* = 0.001) (Table 6). Before the CB period, 91.8% of participants who intended to have a healthy diet were classified into the medium-to-high SR groups whereas during the CB period, 87.7% were in the medium-to-high SR groups and this difference was statistically significant (*p* = 0.011). Importantly, amongst participants who intended to have a healthy diet, 70.9% of them displayed no change or improved eating habits and showed medium-to-high levels of SR during CB.

**Table 6:** Self-regulatory eating behaviour (SR) before and during circuit breaker (CB).

Before CB	During CB N (%)			P value
	Low	Medium	High	
Low	19 (2.9)	26 (4.0)	9 (1.4)	0.001
Medium	50 (7.6)	348 (53.0)	31 (4.7)	
High	12 (1.8)	54 (8.2)	107 (16.3)	

### Association of self-regulatory eating behaviours to stress levels

Participants who reported no change in SR were 2.11 times more likely to be non-stressed as compared to those has deteriorated after adjusting for age, gender, nationality, marital status, having a domestic helper, and number of days spent on moderate-vigorous activity (OR 2.11 95% CI 1.27–3.48, *p* = 0.004) (Table 7). Table 8 demonstrated that HCWs who reported no change in SR have a higher proportion of non-stressed status regardless of the types of occupation. However, it was noted that there were statistical differences between changes in SR and stress levels among nurses (*p* = 0.022) and allied health professionals (*p* = 0.036).

**Table 7:** Association of change in self-regulatory eating behaviours (SR) and stress level using multivariate binary logistic regression and adjusted for age, gender, nationality, marital status, having domestic helper, staying condition and number of days spent on moderate-vigorous activity.

Covariates	Odd ratio, OR (95% CI)	P value
Age	1.05 (1.02, 1.08)	0.001
**Gender**		
Female	Reference	0.447
Male	1.26 (0.69, 2.29)	
**Nationality**		
Citizen/Singapore PR	Reference	0.044
Non-Citizen/non-Singapore PR	2.46 (1.02, 5.89)	
**Marital status**		
Married	1.17 (0.69, 1.98)	0.553
Never married	Reference	
**Change in SR**		
Deteriorated	Reference	
Remain unchanged	2.11 (1.27, 3.48)	0.004
Improved	1.46 (0.68, 3.14)	0.333
**Having domestic helper**		
Yes	Reference	0.004
No	2.13 (1.28, 3.55)	
**Staying condition**		
Living own house	1.00 (0.54, 1.87)	0.985
Living with parents/sibling/relative	Reference	0.383
Rented	0.71 (0.33, 1.53)	
**Changed in number of days spent on moderate-vigorous activity**		
Reduced	Reference	0.164
Unchanged	1.39 (0.87, 2.21)	0.843
Increased	1.06 (0.58, 1.95)

**Table 8:** Distribution of change in self-regulatory eating behaviours (SR) and stress level stratified by healthcare workers (HCW).

	Stress		P value
	Yes N (%)	No N (%)	
**(a) All**			
Change in SR			0.002
Deteriorated	34 (29.3)	82 (70.7)	
Remain unchanged	72 (15.2)	402 (84.8)	
Improved	13 (19.7)	53 (80.3)	
**(b) Doctor**			
Change in SR			0.623
Deteriorated	2 (25.0)	6 (75.0)	
Remain unchanged	6 (14.0)	37 (86.0)	
Improved	2 (25.0)	6 (75.0)	
**(c) Nurse**			
Change in SR			0.022
Deteriorated	12 (40.0)	18 (60.0)	
Remain unchanged	28 (17.1)	136 (82.9)	
Improved	5 (20.8)	19 (79.2)	
**(d) Allied health professional**			
Change in SR			0.036
Deteriorated	12 (27.9)	31 (72.1)	
Remain unchanged	21 (11.9)	155 (88.1)	
Improved	3 (15.8)	16 (84.2)	
**(e) Administrative staff**			
Change in SR			0.871
Deteriorated	8 (22.9)	27 (77.1)	
Remain unchanged	17 (18.7)	74 (81.3)	
Improved	3 (20.0)	12 (80.0)	

## Discussion

Psychological distress during the Covid-19 pandemic has been shown to adversely affect both HCWs’ mental and physical health, decreasing performance and efficiency at work. The CB limited the movement and interactions in the community, resulting in a drastic change in the daily activities of the general public. Healthcare workers (HCWs) needed to stay committed to caring for the population while being subjected to the restrictions themselves. Many studies explored how CB measures were associated with reduced physical activities and poorer dietary habits^8,11,13,16^ during the CB period. A number of these studies described the importance of healthy lifestyle behaviours—having sufficient exercise and a better choice of diet were linked to better mental outcomes and reduced negative moods such as stress^11,13,17^. Prioritising the quality of the diet has great influence on the HCWs^18^, especially during the response period with heavy work intensity coupled with physical and mental exhaustion, in addition to a decrease in physical activities, sleep problems and changes in dietary habits and body weight^19^. A systematic review of longitudinal studies conducted by González-Monroy et al. provided a comprehensive overview of eating behaviour characteristics associated with COVID-19 pandemic. Our study outcome is consistent with this and other reviews as several outcomes such as overeating and the influence of unhealthy food choices during the pandemic have been observed^23^. Emotional eating is defined as overeating after stress and negative emotions and people tend to eat as a mechanism to cope with mood changes^24^. A study conducted by Arora and Grey, revealed that the consumption of food with a short shelf life such as fresh vegetables, fruits, meat, chicken, and fish were decreased. The consumption of highly processed food with long life rich in fat, sugar and salt were increased^24^ as shown in the study, consistent with our survey results.

Regular exercise and positive eating habits with positive consequences on the mental well-being were vital^22,25^ to ensure that frontline HCWs stood resilient and in good health to perform their daily mission in healthcare. Herein our study, we wanted to find out how Singapore HCWs coped with the CB in terms of their health and lifestyle behaviours. We aimed to correlate their self-regulatory eating behaviours (SR) and determine how SR affected their mental well-being. Self-regulatory dietary temptation had been demonstrated in a randomized control trial to be as effective as dietary and physical activity advice in terms of weight loss and related outcomes^26^. Furthermore, SR as a form of behavioural therapy for weight loss also improved physical activity levels^27^ and motivation to exercise^24,28^. Unsurprisingly, with improved SR and physical activity levels, improved mood was observed, and this ensured healthy behaviours and sustained weight reduction^28^. Our findings suggested that an individual’s SR may be important to assess an individual’s self-perception of well-being in terms of stress—having no change in SR before and during the CB appears to be at increased odds of being not stressed. Furthermore, comparison amongst the ethnicities of our participants and the job scopes also revealed differences that were statistically significant and suggested the relevance of different intervention lifestyle programmes tailored for specific groups of HCWs of varying profiles. In our study, we noted that nurses and allied health professionals had statistically different changes in SR before and during the CB. One study reported that psychological distress was strongly associated with emotional eating^29^. Based on our study, identification of at-risk occupational groups with potential high stress levels through assessment of self-regulatory eating behaviours would prioritise plans for targeted interventions to manage cues to prevent overeating and stress. The maintenance and improvement of self-regulatory behaviours were likely to indicate an individual’s ability to cope better with stress^30^. In addition to providing HCWs with tips on making better food choices, healthy lifestyle intervention programmes can focus on self-management strategies through empowering HCWs to set measurable and realistic goals to self-regulate their eating behaviours. Consumption of a healthy diet and participation in physical activity^21^ was essential to positively influence their health and well-being, this conclusion is similar to another study on long-term care workers from Canada^31^, whereby workers who engaged with health-conscious behaviours were able to maintain good emotional health^31–33^. These findings supported the novelty of our study which proposed that changes or no change in SR could be a valuable indication of no stress. Our study demonstrated that changes in SR as an independent variable that would reflect the stress levels and likely coping strategies of HCWs in tumultuous times.

While our study sample was representative of occupational distribution in the healthcare institutions surveyed, we were unable to determine the total outreach of the survey due to the limitations of the dissemination methods. Therefore, we were unable to ascertain the representativeness of the survey response rates^31^ as well as the possibility of selection bias as only individuals who were interested and/or had access to the survey online would participate. As this study focused on reporting by HCWs in public healthcare institutions, it might not be generalizable to general populations or HCWs in different healthcare settings. As this was a cross-sectional study design, causality could not be made on the relationship between changes in SR and stress levels. Our measures of SR, dietary choices and habits were based on self-reporting, and this could introduce recall bias. Despite the limitations discussed, our study revealed novel findings on the temporal changes (before and during CB) in SR on mental well-being in terms of stress levels in HCWs.

## Conclusion

We reported that maintenance of self-regulatory eating behaviour in HCWs was associated with individuals who were less likely to be moderately or severely stressed. The pertinent point to note was despite the obvious changes in dietary choices available in addition to the restrictions imposed, HCWs who continued to maintain pre-CB SR were more likely to be stress-free and cope with the adversities. Therefore, more research needs to be done to explore health behaviours in highly stressful occupations such as HCWs and workplace health promotion interventions should continue to implement occupational risk prevention^31^, especially in difficult moments such as the global COVID-19 pandemic. Short screening questionnaires based on SR changes should be developed and explored as surveillance tools for assessment of HCWs’ general well-being such that personalized interventions to vulnerable groups of workers could be implemented effectively on the ground. It would be ideal if the interventions were participatory and inclusive of HCWs through a bottom-up approach as opposed to exclusively top-down approaches^34,35^.

## Supplementary Information

Supplementary Table S1.
