# Supplementary material for: Healthcare workers’ self-regulatory eating behaviours are associated with being stress-free during the Covid-19 lockdown in Singapore
Source: Sci Rep. 2022 Sep 28;12:16257. doi: 10.1038/s41598-022-19001-1 (PMC9518944; doi:10.1038/s41598-022-19001-1)
Supplement: Supplementary file 1 — Supplementary Table S1. [file 41598_2022_19001_MOESM1_ESM.docx]

Supplementary Tables: Table S1A and S1B Items adapted from SREBQ (14) to assess for self-regulatory eating behaviours (SR) and scoring system to determine low/medium/high SR.

Table S1A

| Item | Interpretation |
| --- | --- |
| A. I give up too easily on my eating intentions | Ability to stick to eating intentions and continuously work towards long term capacity to self-regulate eating behaviors |
| B. I am good at resisting tempting food | Ability to control behaviour, thoughts, feeling, attention and eat in accordance with your intentions/ short term capacity to regulate eating behaviours |
| C. I easily get distracted from the way I intend to eat | Ability to control thoughts and attention and keep eating goals in mind |
| D. If I am not eating in the way I intend, I make changes | Ability to compare actual behavior to eating intentions (reference) and make adjustments when necessary to achieve intention |
| E. I find it hard to remember what I have eaten throughout the day | Ability to monitor and be aware of actual eating behaviors |

| Items | Never | Rarely | Sometimes | Often | Always |
| --- | --- | --- | --- | --- | --- |
| A, C & E | 5 | 4 | 3 | 2 | 1 |
| B&D | 1 | 2 | 3 | 4 | 5 |
| Mean score (Adding A+B+C+D+E/5) | | | Level of self-regulation (SR) | | |
| <2.8 | | | Low | | |
| 2.8 – 3.6 | | | Medium | | |
| >3.6 | | | High | | |

Table S1B
